# Application of CFS‐Loaded CMC/GEL Spray Coating for the Small Fruit Blueberries Preservation

**DOI:** 10.1002/fsn3.72134

**Published:** 2026-07-28

**Authors:** Lanlan Wei, Ziyi Qin, Sichen Li, Congsheng Yan

**Affiliations:** ^1^ College of Food Engineering Anhui Science and Technology University Chuzhou China; ^2^ Key Laboratory Horticultural Crop Germplasm Innovation and Utilization (Co‐Construction by Ministry and Province), Institute of Horticulture Anhui Academy of Agricultural Science Hefei China

**Keywords:** blueberry, cell‐free supernatants, functional spray, preservation, small fruit

## Abstract

Blueberries are highly susceptible to decay due to mechanical damage such as bruising or impact. Traditional postharvest preservation methods such as brush and dip coating generate solvent waste, incur high labor costs, and may also leave visible brush marks. Moreover, due to their “one‐by‐one” coating process, which lowers the processing efficiency, these traditional methods are not suitable for small fruits such as blueberries. To solve this problem, a functional spray was developed in this work using gelatin (GEL) and sodium carboxymethyl cellulose (CMC) as the matrix, and a cell‐free supernatant (CFS), produced by the centrifugation of lactic acid bacteria fermentation broth, was used as the active ingredient. Microstructural (SEM), intermolecular interaction (FTIR), and crystal structure (XRD) analyses of a CFS/CMC/GEL composite film demonstrated that the components of the functional spray had good compatibility. Moreover, this film had a greater water vapor permeability (WVP), reduced transmittance, and enhanced light‐blocking properties compared with a CMC/GEL film. The 100% CFS/CMC/GEL functional spray exhibited improved antimicrobial performance against *Escherichia coli* , *Staphylococcus aureus* , *Listeria monocytogenes* , and *yeast*. Finally, a blueberry preservation experiment indicated that the blueberries sprayed with 100% CFS/CMC/GEL exhibited a lower decay rate, reduced weight loss, and lower malondialdehyde (MDA) level compared with the control group, whereas total soluble solids (TSS) and titratable acidity (TA) levels were higher. Notably, the firmness of the blueberries did not significantly change. These spray coating preservation tests demonstrate that the CFS/CMC/GEL composite coating effectively extends the shelf life of small fruits such as blueberries.

## Graphical abstract

Schematic illustration showing the fabrication of the CFS‐loaded CMC/GEL composite spray coating and its application in the blueberry preservation. The preservation experiment indicated that the blueberries sprayed with 100% CFS/CMC/GEL exhibited a lower decay rate, reduced weight loss, and lower malondialdehyde (MDA) level compared with the control group, whereas total soluble solids (TSS) and titratable acidity (TA) levels were higher.

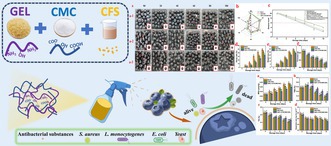

## 1 Introduction

Blueberries are widely consumed for their unique flavor and abundant anthocyanins, ellagic acid, and vitamin C, which provide antioxidants and contribute to lipid‐lowering and other health benefits (Wang et al. 2017). However, blueberries also exhibit high respiration rates, susceptibility to mechanical damage, and vulnerability to microbial contamination, shortening their postharvest shelf life (Tang et al. 2023). Traditional postharvest preservation techniques include spraying, fumigating, and soaking with various chemicals such as ClO_2_, organic acids, CaCl_2_, and 1‐methylcyclopropene, but most of these agents have raised consumer concerns about residues (Wang et al. 2020). Therefore, the development of an efficient, eco‐friendly, and antimicrobial method would be highly desirable for the field of blueberry preservation.

Bio‐preservation is an emerging technology that extends food shelf life by utilizing the antimicrobial activity of microorganisms (bacteria, molds, and yeasts) and their metabolites (Akman et al. 2023). Compared to the utilization of chemical preservatives, bio‐preservation can better preserve food quality under mild conditions, offering enhanced safety and environmental benefits. Lactic acid bacteria (LAB), which are good for human health and are regarded as the most widely used probiotics (Stabnikov et al. 2025), show good promise as natural substitutes for chemical preservatives in food applications due to their safety and functional characteristics (Myktybayeva et al. 2024). Notably, LAB are particularly promising due to their key metabolite, cell‐free supernatant (CFS), which contains bioactive compounds such as oligosaccharides, short‐chain fatty acids, vitamins, enzymes, phenolics, and organic acids (Akman et al. 2023). The antimicrobial effects of CFS are caused by the synergistic action of organic acids, hydrogen peroxide, and bacteriocin‐like peptides, which inhibit microbial growth by affecting cellular physiology, enzyme activity, protein synthesis, nutrient uptake, and membrane permeability (Shafipour‐Yordshahi et al. 2020). Additionally, the metabolites of LAB can inhibit browning‐related enzymes such as polyphenol oxidase (PPO) and peroxidase (POD), extending the shelf life of fruits and vegetables (Gajendran and Rajamani 2024). When applied as a food preservation agent, LAB metabolites such as diacetyl and exopolysaccharides also help reduce moisture loss and maintain nutritional stability, further improving preservation efficacy (Yépez et al. 2017). In a study on bread preservation, the CFS of *Lactobacillus sakei PLL12* (*L. sakei PLL12*) and *Lactococcus lactis PLL15* ( *L. lactis* *PLL15*) effectively inhibited spore germination, disrupted the integrity of fungal cell membranes and cell walls by inhibiting the synthesis of ergosterol, and generated reactive oxygen species (ROS), which attacked mitochondria. This induced apoptosis, extending the shelf life of bread by 2–3 days (Zhang et al. 2026).

Coating preservation techniques combine polysaccharides, proteins, or starches with cross‐linking agents and antibacterial agents to prepare a preservation coating solution, which is applied to fruit and vegetable surfaces to form a dense film. This coating regulates the internal atmosphere and reduces moisture loss, and the antibacterial agents inhibit microbial growth to extend the food shelf life (Abdel Aziz and Salama 2022). Although traditional coating methods such as dip and brush coating demonstrate some effectiveness, several limitations persist. Dip coating processes require large containers and fixed equipment, and they have poor operational flexibility. Moreover, thick and uneven coatings are often formed, and solution wastage always occurs due to gravitational drainage (Andrade et al. 2012). Brushing methods heavily rely on manual labor, and this method often leaves treated food with brush marks and an unevenly applied coating. Therefore, a professional operation is typically required to achieve a smooth and uniform coating (Wang et al. 2023). Spraying is considered to be a novel coating strategy for food preservation. In a spraying process, the coating solution is dispersed into droplets by a specialized nozzle and evenly sprayed to form a preservation film (Han et al. 2016). Compared with traditional coating preservation methods, spraying processes have significant advantages, including greater flexibility and easier operation under various environments. Moreover, spraying generates fast‐drying coatings, leading to improved efficiency and a reduced risk of microbial cross contamination (Denis et al. 2015). Therefore, spraying has become a key technology for maintaining the postharvest quality of small fruits, such as blueberries and cherries, and it holds broad application prospects (Olmedo et al. 2025).

As a carboxymethylated derivative of cellulose, sodium carboxymethyl cellulose (CMC) is extensively utilized in food packaging coatings owing to its high viscosity, clarity, non‐toxicity, biodegradability, and superior film‐forming properties (Li et al. 2020). However, pure CMC films typically exhibit poor performance. Therefore, CMC is commonly blended with other film‐forming matrices to obtain films with enhanced properties. Due to its biodegradability, ease of processing into films, and good mechanical properties, gelatin (GEL) is frequently combined with CMC to prepare CMC/GEL composite films that exhibit improved flexibility, tensile strength, puncture resistance, and thermal stability, along with a reduced elongation at break (Cai et al. 2022). Other materials can also be incorporated into these composite films. For example, in a study evaluating films composed of curcumin, GEL, and CMC, the addition of curcumin was reported to significantly increase film thickness, mechanical properties, and antibacterial activity (Lv et al. 2025). Consequently, when these films were applied as food coatings, the shelf lives of grapes and bananas were extended to 9 and 6 days, respectively. Pure CMC/GEL solutions often exhibit excessive viscosity due to polymer chain entanglement, which leads to nozzle clogging and uneven film formation. This has limited their practical utilization in spraying applications (Liu et al. 2024). However, biosurfactants, such as the glycolipids and lipopeptides in CFS, can effectively reduce the surface tension of these solutions to promote better droplet spreading and adhesion on fruit surfaces (Gayathiri et al. 2023; Deng et al. 2023). Furthermore, the exopolysaccharides (EPS) in CFS can act as natural stabilizers and emulsifiers, ensuring the smooth spraying and uniform dispersion of composite solutions (Jiang et al. 2022).

In this study, a bio‐based spray coating was utilized to effectively prolong the shelf life of blueberries. GEL and CMC served as the functional spray matrix, while CFS was incorporated to enhance the bioactivity of the coating. The composite films were characterized by SEM, FTIR, and XRD, and their WVP, optical properties, and antimicrobial activity were evaluated. In parallel, the effects of the functional spray on blueberry preservation were assessed during storage. This work proposes an eco‐friendly preservation strategy and demonstrates the use of cell‐free supernatant in a functional spray, offering both theoretical and practical value.

## 2 Materials and Methods

### 2.1 Materials and Reagents

Sodium carboxymethyl cellulose (CMC) and gelatin (GEL) were purchased from Shanghai Chemical Industry Park Co. Ltd. *Lactiplantibacillus plantarum ATCC 7469* (*L. plantarum ATCC 7469*), *Escherichia coli ATCC 25922* (*E. coli ATCC 25922*), *Staphylococcus aureus ATCC 6538* (*S. aureus ATCC 6538*), *yeast ATCC9763*, and *Listeria monocytogenes CICC 21622* (*L. monocytogenes CICC 21622*) were acquired from Shanghai Gaoxin Chemical Glass Instrument Co. Ltd. Lactic acid bacteria culture medium (DeMan, Rogosa, Sharpe, MRS), potato‐dextrose agar, Luria‐Bertani broth, and agar were purchased from Shanghai Luwei Technology Co. Ltd. Trichloroacetic acid (C_2_Cl_3_NaO_2_), thiobarbituric acid (C_4_H_4_N_2_O_2_S), sodium hydroxide (NaOH), oxalic acid (H_2_C_2_O_4_), barium chloride (BaCl_2_), and phenolphthalein (CHO_4_) were acquired from Shanghai Maclean Biochemical Co. Ltd. Fresh blueberries were purchased from a local supermarket in Chuzhou, Anhui Province, China.

### 2.2 Preparation of CFS/CMC/GEL Spray Solution

A 5% (v/v) bacterial suspension was transferred to MRS broth and incubated at 37°C for 48 h. After incubation, the culture was centrifuged at 5000 rpm for 20 min at 4°C, and the supernatant was subsequently filtered through a 0.22 μm membrane filter to obtain CFS. The CFS total acidity (TA) was determined by titrating the sample with 0.02 mol L^−1^ NaOH to pH 8.2 and the TA of CFS was 1.46 ± 0.03 (% Lactic acid). In addition, after being calibrated, the pH of CFS was measured by the pH meter (Mettler Toledo, FE28) and the pH was 4.00 ± 0.02. Finally, the filtrate was stored at 4°C for further use.

To prepare the CMC/GEL spray solution, CMC (0.8 g) and GEL (0.8 g) were weighed and dissolved in 100 mL distilled water, then magnetically stirred for 3 h. The distilled water was partially or completely replaced by 50 mL, 75 mL, or 100 mL CFS to prepare 50% CFS/CMC/GEL, 75% CFS/CMC/GEL, and 100% CFS/CMC/GEL spray solutions. After their preparation, 10 mL of each spray solution was transferred to a Petri dish and dehydrated at 40°C for 18 h in an incubator for subsequent characterization.

### 2.3 Scanning Electron Microscopy

Surface morphology was examined by scanning electron microscopy (SEM, Supra 55; Zeiss) at an accelerating voltage of 20 kV. Samples were mounted on specimen holders and gold‐coated in a vacuum evaporator prior to analysis.

### 2.4 Fourier Transform Infrared Spectroscopy

The Fourier transform infrared (FTIR) spectra of the whole films were acquired with a Nicolet iS10 instrument (Thermo Fisher) in the range of 500–4000 cm^−1^ with a resolution of 2.0 cm^−1^. Each sample was combined with KBr and thoroughly ground in an agate mortar prior to analysis.

### 2.5 X‐Ray Diffraction

X‐ray diffraction (XRD) was conducted on a diffractometer (Beijing Purkinje General) at 36 kV and 24 mA within the 2θ scanning range of 5°–50° using a scan rate of 5° min^−1^.

### 2.6 Color and Optical Properties

The colors of the film were analyzed with a 3nh colorimeter (3nh Technology Co. Ltd.). The lightness (L*), redness‐greenness (a*), and yellowness‐blueness (b*) of the whole composite films were measured at five randomly selected points. During measurement, each film was placed on a standard white background (L* = 95.10, a* = 0.28, b* = 1.89), and the color parameters were recorded.

(1) $$∆E=\sqrt{{\left({L^{*}}_{\text{film}}-{L^{*}}_{\text{standard}}\right)}^{2}+{\left({a^{*}}_{\text{film}}-{a^{*}}_{\text{standard}}\right)}^{2}+{\left({b^{*}}_{\text{film}}-{b^{*}}_{\text{standard}}\right)}^{2}}$$

The light transmittance of the films was determined at 600 nm using a UV–visible spectrophotometer (UV‐1800, Shimadzu). Prior to measurement, the films were cut into 40 mm × 10 mm samples and placed in cuvettes, and an empty cuvette was used as the blank control (Jakubowska et al. 2023). Each sample was measured in triplicate, and the average value was reported. The opacity of each film sample was calculated according to Equation (2):

(2) $$\begin{array}{c} \text{Opacity}=\frac{-\log T_{600}}{x} \end{array}$$

where *x* denotes the film thickness (mm) and T_600_ denotes the fractional transmittance at a wavelength of 600 nm.

### 2.7 Water Vapor Permeability

The water vapor permeability (WVP) of each film was determined by adding anhydrous calcium chloride (CaCl_2_) to a permeation cup, which was sealed with a sample film and weighed (Zhang et al. 2022). The film thickness was measured with a vernier caliper. Next, the sealed permeation cup was placed in a desiccator containing water at the bottom. Under the controlled conditions of 75% relative humidity and 25°C, the change in mass (Δ *m*) was recorded once the mass gain rate was < 5%. The WVP was then calculated by using Equation (3):

(3) $$\begin{array}{c} \mathrm{WVP}\;\left(g\cdot \mathrm{mm}/cm^{2}\cdot h\cdot \mathrm{Pa}\right)=\frac{∆m\times d}{A\times ∆t\times \text{∆}p} \end{array}$$

where Δ*m* represents the weight gain of the permeation cup (g), *d* is the average membrane thickness (mm), *A* is the transfer area (9 cm^2^), Δ*t* is the measured time interval (2 h), and ∆*P* denotes the water vapor pressure difference across the membrane (3.168 kPa).

### 2.8 Antibacterial Activity Analysis

100 μL *Staphylococcus aureus* , *Listeria monocytogenes* , *Escherichia coli* , and *yeast* bacterial suspensions were evenly spread onto solid media. Then, holes were punched using a 6 mm sterilized puncher, and a 100 μL volume of the CFS/CMC/GEL solution was added into each hole (Li et al. 2023). *S. aureus* , *L. monocytogenes* , and *E. coli* were incubated at 37°C for 24 h, while *yeast* was incubated at 28°C for 48 h. Three parallel experiments were conducted for each sample, and the diameter of the inhibition zone was recorded.

## 3 Application of Coating Solutions for Preservation of Blueberries

**FIGURE 1:**
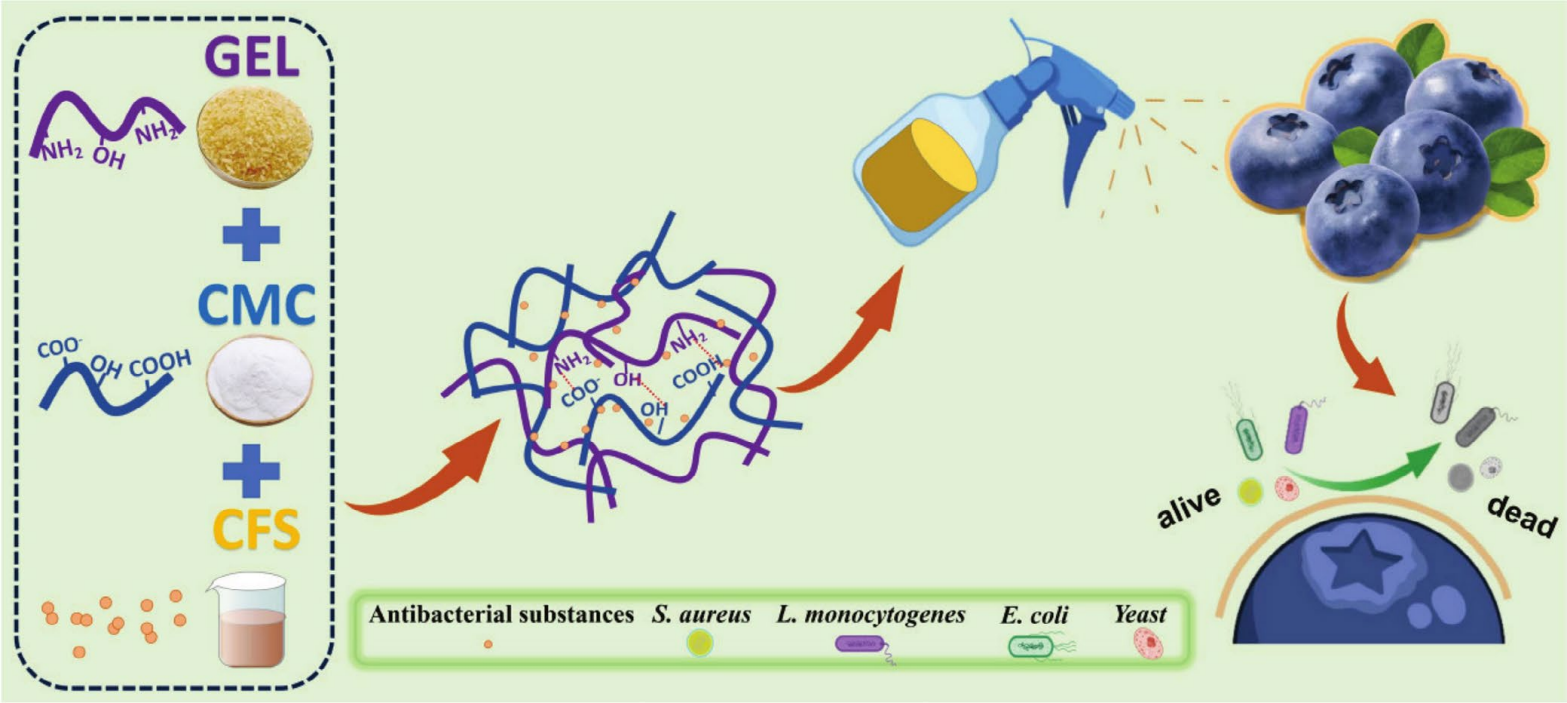
Schematic illustration of functional spray preparation and application in the preservation of blueberries.

The preparation of the functional sprays and their application in blueberry preservation are illustrated in Figure 1. Driscoll's blueberries (≈12 mm diameter, uniform shape) at commercial maturity with a full blue color, firm texture, and no visible defects or damage were selected. The functional spray was applied by using a manual pressure sprayer (nozzle diameter: 1.0 mm) with a flow rate of 50 mL min^−1^ and a spray pressure of 0.1 MPa. A spraying distance of 15–20 cm was maintained between the nozzle and the fruit surface. For each group (consisting of 30 g blueberries), 20 mL of coating solution was used. Groups A, B, and C were sprayed with distilled water, CMC/GEL, and 100% CFS/CMC/GEL spray, respectively. The coated samples were then stored under ambient conditions (temperature of 26°C and relative humidity of 55%). All analytical measurements were performed every 2 days in triplicate, and the results were expressed as the mean ± standard deviation (SD).

### 3.1 Blueberry Quality

#### 3.1.1 Sensory Evaluation of Blueberries

Photographs were taken every 2 days to record the blueberry spoilage. A panel of 10 randomly selected people was trained to assess the samples with a sensory evaluation scale (Cheng et al. 2024), as shown in Table 1.

**TABLE 1:** Sensory and quality evaluation of blueberries.

Score range	Flavor description	Appearance	Flesh texture
8–10	Rich, characteristic blueberry flavor with no off‐flavors	Plump, uniform, glossy berries with bright color; no decay or defects	Firm, elastic flesh, juicy, close to freshly harvested
6–8	Normal blueberry flavor, acceptable	Good appearance, uniform size and color, no visible decay, slight dullness	Slightly less firm than freshly harvested; still acceptable texture with minor elasticity loss
4–6	Mild, slightly bland flavor	Dull surface, minor blemishes or lesions, less attractive appearance	Slightly soft, reduced crispness; juiciness loss but intact structure
2–4	Poor flavor, noticeable off‐flavor	Slight shriveling, uneven color, minor decay	Soft, fragile flesh, slight breakage, partially collapsed structure
0–2	Strong off‐odor, rotting smell, unacceptable	Severely shriveled, decayed, discolored, visible defects	Mushy, sticky texture, unstructured flesh, tissue integrity lost

#### 3.1.2 Weight Loss

The blueberry weight loss during storage was calculated using Equation (4):

(4) $$\begin{array}{c} \text{Weight loss rate}\;\left(\%\right)=\frac{W_{0}-W_{Z}}{W_{0}}\times 100\% \end{array}$$

where *W*_0_ and *W*_Z_ represent the blueberry weights before and after storage, respectively. Values were averaged over three independent replicates.

#### 3.1.3 Decay Rate

For each treatment group, 30 blueberries were randomly selected to assess the decay rate. Fruits exhibiting visible signs of spoilage, such as mold growth, juice leakage, collapse, or skin cracking, were classified as decayed fruits. The decayed fruits were counted at 2‐day intervals, and the decay rate was calculated using Equation (5):

(5) $$\begin{array}{c} \text{Decay rate}\;\left(\%\right)=\frac{\text{Number of decayed fruits}}{\text{Total number of fruits}}\times 100\% \end{array}$$

#### 3.1.4 Firmness

For each group, the firmness of 10 randomly selected blueberries was measured using a texture analyzer (CT3 Texture Analyzer, Brookfield, USA). A cylindrical probe with a 2.0 mm diameter (TA39) was used to penetrate the surface of each blueberry with a speed of 2.0 mm s^−1^, a penetration depth of 6 mm, and a trigger force of 5.0 g. Each blueberry was tested three times, and the mean value was obtained. The calyx end of each blueberry was avoided when performing the firmness test.

### 3.2 Determination of Malondialdehyde, Titratable Acidity, and Total Soluble Solids

#### 3.2.1 Malondialdehyde Content

The malondialdehyde (MDA) content of the blueberries during storage was determined via the thiobarbituric acid (TBA) method (Jiang et al. 2019). 1.0 g blueberry sample was homogenized with 5.0 mL of trichloroacetic acid (TCA) and centrifuged at 10,000 rpm for 10 min. Subsequently, 2.0 mL of the supernatant was mixed with 2.0 mL of TBA solution and centrifuged again. The supernatant absorbance was measured at 450, 532, and 600 nm, and the MDA concentration was calculated using Equation (6):

(6) $$\begin{array}{c} \mathrm{MDA}\;\left(\%\right)=\frac{\left[6.45\times \left({\mathrm{OD}}_{532}-{\mathrm{OD}}_{600}\right)-0.56\times {\mathrm{OD}}_{450}\right]\times V}{\;V_{S}\times m\times 1000}\;\left(\text{μmol}\;g^{-1}\right) \end{array}$$

where *V* represents the total volume of the sample extract (mL), *V*_s_ represents the volume of the filtrate used for analysis (mL), and *m* is the sample mass (g).

#### 3.2.2 Titratable Acidity Content

To determine the titratable acidity (TA) content, a 10 g blueberry sample was homogenized, and 20 mL of the resulting filtrate was collected. Then, 1–2 drops of phenolphthalein indicator were added, and titration was performed with 0.01 mol L^−1^ NaOH until a faint pink color persisted for 30 s. The NaOH volume was then recorded. Distilled water was used as a blank control. The TA content was then calculated using Equation (7):

(7) $$\begin{array}{c} \mathrm{TA}\;\left(\%\right)=\frac{V\times c\times \left(V_{1}-V_{0}\right)}{V_{s}\times m}\times 100\% \end{array}$$

where *V* denotes the total volume of the filtrate (mL), *V*_s_ is the filtrate volume used for titration (mL), *c* is the concentration of the NaOH solution (mol L^−1^), *V*_1_ is the NaOH volume consumed during sample titration (mL), *V*_0_ is the NaOH volume consumed during blank titration (mL), and m denotes the sample mass (g).

#### 3.2.3 Total Soluble Solids Content

To determine the total soluble solids (TSS) content, a 5.0 g blueberry sample was randomly selected, homogenized, and filtered to obtain the blueberry juice. The TSS content was then determined by a handheld refractometer (3nh Technology Co. Ltd., Shenzhen, China).

#### 3.2.4 Respiration Rate

The respiration rate was measured by the method reported by Huang et al. (2022) using a sealed glass desiccator. This desiccator consisted of a glass cylinder, a curved glass top with a handle, and an internal white ceramic porous partition. The frosted contact area between the cylinder mouth and the lid was coated with Vaseline to achieve complete airtight sealing. For analysis, 0.25 kg of blueberries was placed in the sealed glass desiccator and incubated with 10 mL of 0.4 mol L^−1^ NaOH for 0.5 h. Subsequently, 5.0 mL of saturated BaCl_2_ was added, and the mixed solution was titrated with 0.2 mol L^−1^ oxalic acid using phenolphthalein as an indicator. Finally, the respiration rate was calculated using Equation (8):

(8) $$\text{Respiration rate}\;\left(\mathrm{mg}\;{\mathrm{CO}}_{2}\;{\mathrm{kg}}^{-1}\;h^{-1}\right)=\frac{\left(V_{1}-V_{2}\right)\times c\times 22}{m\times t}$$

where *V*_1_ and *V*_2_ respectively represent the volumes of oxalic acid solution consumed by the blank and the sample groups (L), *c* is the molar concentration of oxalic acid (mol L^−1^), *m* denotes the sample mass (kg), *t* is the duration of the respiration measurement (h), and the mass conversion coefficient for CO_2_ is 22.

### 3.3 Statistical Analysis

The data were analyzed using IBM SPSS Statistics (version 27.0.1) and Origin 2018. One‐way analysis of variance (ANOVA) followed by Duncan's multiple range test was employed, and the Kruskal–Wallis test was used for non‐parametrically distributed data. The statistical significance level was set at *p* < 0.05, and the results are presented as means ± standard deviation (SD).

To quantify the decay in sensory quality during storage, the sensory evaluation scores of each group collected at different storage days were analyzed by a zero‐order kinetic model. The *R* ^2^ value was adopted to evaluate the fitting degree of the zero‐order model. A sensory score of 6 was defined as the sensory failure threshold, representing the acceptable limit of overall sensory quality for consumers. Each sensory evaluation score was calculated using Equation (9):

(9) $$\begin{array}{c} A_{t}=A_{0}-k_{0}t \end{array}$$

where *A*_t_ is the sensory evaluation score at storage time *t* (day), *A*_0_ is the initial sensory score at Day 0, *k*_0_ is the zero‐order degradation rate constant, and *t* is the storage time (day).

## 4 Results and Discussion

### 4.1 SEM Analysis

**FIGURE 2:**
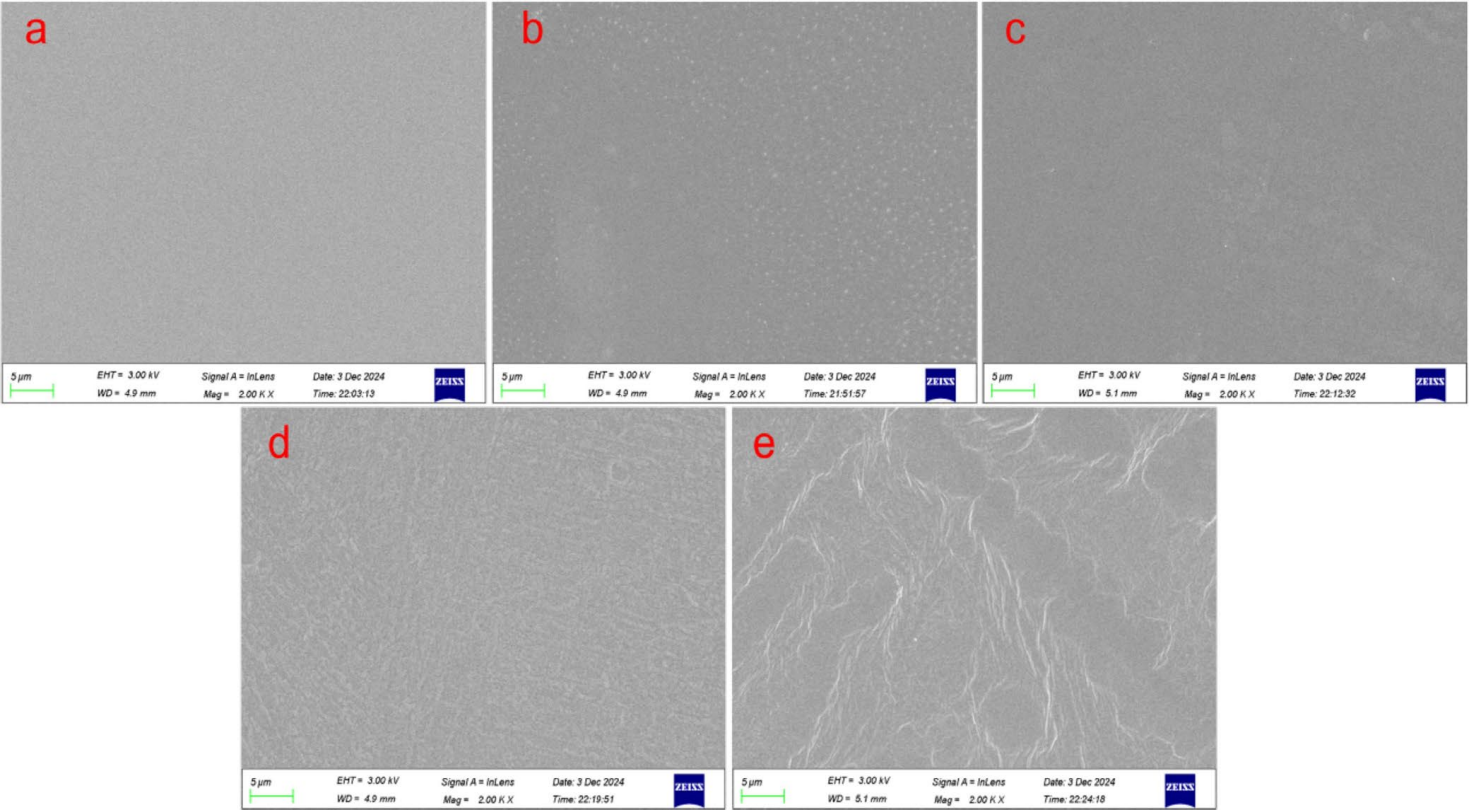
SEM images of CMC (a), CMC/GEL (b), 50% CFS/CMC/GEL (c), 75% CFS/CMC/GEL (d), and 100% CFS/CMC/GEL (e).

The microstructure and compatibility of the prepared films were examined by SEM, as shown in Figure 2a–e. The pure CMC film (Figure 2a) exhibited a smooth and compact surface without obvious pores or cracks. The surface texture of the composite CMC/GEL film (Figure 2b) was uniform and smooth, with no signs of aggregation. This was possibly ascribed to hydrogen bonding between the molecules in this film, which would facilitate the formation of a homogeneous mixture of components (Lv et al. 2025). Compared with the CMC/GEL film, the surfaces of the CFS/CMC/GEL composite films became rougher and more rigid with increasing CFS concentration. This was potentially due to the agglomeration of organic salts in the CFS (such as highly crystalline calcium lactate) and residual inorganic salts from the culture medium (Kiran‐Yildirim et al. 2018; Hayek et al. 2019), which would increase the overall surface roughness. Notably, this micro‐roughness matches the natural micro‐nano surface structure of blueberry skin, helping the coating evenly spread and strongly adhere to the fruit surface (Huang et al. 2022).

### 4.2 FTIR and XRD Analysis

**FIGURE 3:**
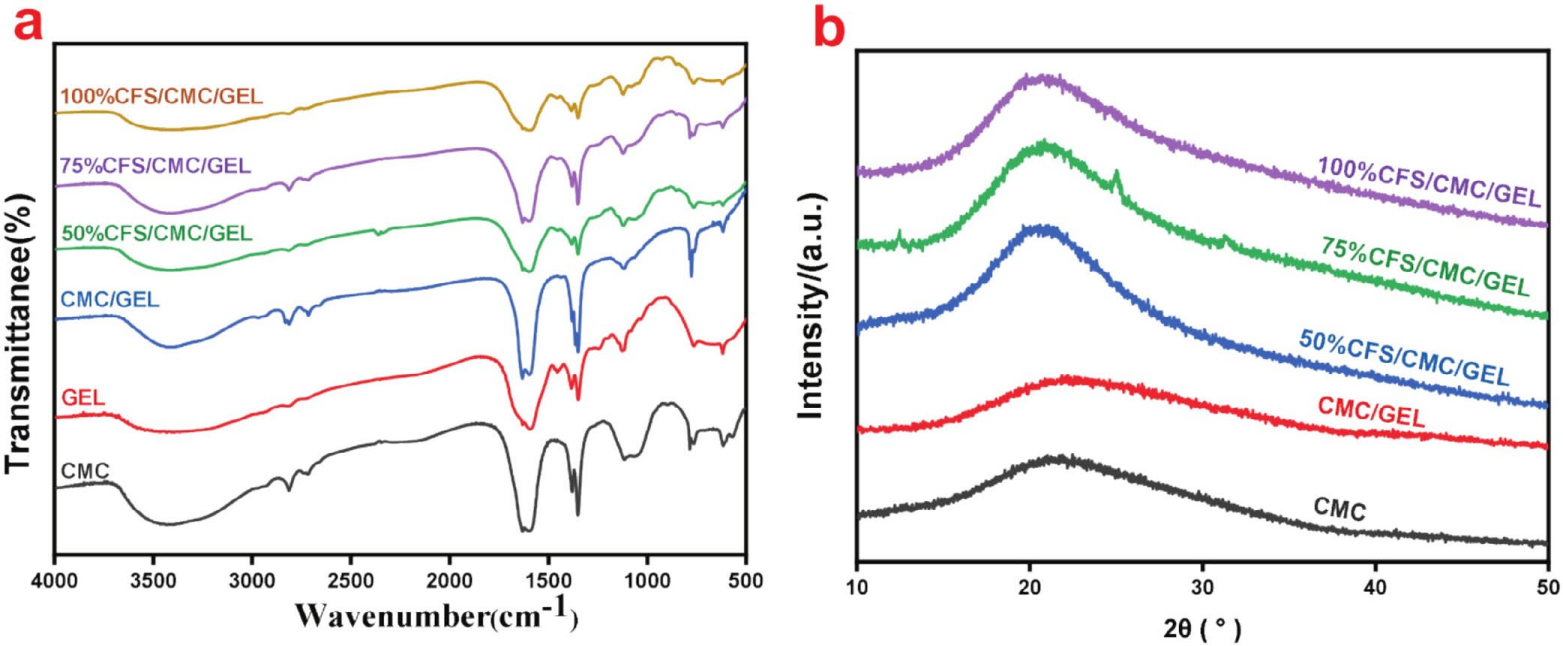
FTIR spectra (a) and XRD patterns (b) of CMC, GEL, CMC/GEL, 50% CFS/CMC/GEL, 75% CFS/CMC/GEL, and 100% CFS/CMC/GEL.

The FTIR spectra of CMC, GEL, CMC/GEL, and the 50%, 75%, and 100% CFS/CMC/GEL samples are shown in Figure 3a. The spectrum of CMC displayed a typical absorption band at 3491 cm^−1^, which was assigned to –OH stretching vibrations. Meanwhile, the peaks at 2930 and 2882 cm^−1^ were ascribed to the symmetric and asymmetric stretching vibrations of CMC (Bhutto et al. 2003). Absorption bands at 1590 and 1415 cm^−1^ were attributed to the stretching vibrations of carboxyl groups, while those at 1035 and 924 cm^−1^ were ascribed to ether linkages typical of polysaccharide structures (Nikonenko et al. 2000). In the FTIR spectrum of GEL, a prominent absorption peak at 1643 cm^−1^ was associated with the amide I band, indicating the secondary structure of the protein backbone (Nur Hazirah et al. 2016). The amide II band at 1560 cm^−1^ represented N–H and C–H bending vibrations, and the amide III band at 1238 cm^−1^ was related to in‐plane N–H and C–H vibrations. The CMC/GEL composite film was formed through electrostatic interactions, without the formation of new chemical bonds (Devi et al. 2012). Therefore, the FTIR spectrum of CMC/GEL displayed the characteristic bands of both components, including peaks at 2974 and 2882 cm^−1^ (–CH stretching) as well as bands at 1035 and 924 cm^−1^ (glycosidic linkages). The FTIR spectra of the CFS/CMC/GEL films did not show any new peaks, suggesting that the chemical structure of the CMC/GEL was unchanged by the addition of CFS. However, as the CFS content increased from 50% to 100%, the –OH stretching band (3200–3500 cm^−1^) broadened and slightly shifted to a lower wavenumber (Figure 3a), indicating stronger hydrogen bonding between the CFS components and the –OH/–NH groups of CMC and GEL (Ma et al. 2020).

The XRD patterns of the prepared films are shown in Figure 3b, with a narrower diffraction peak typically indicating higher atomic ordering and enhanced crystallinity (Morales‐Santiago et al. 2023). CMC showed an intense diffraction peak near 2θ = 20°, corresponding to the crystalline structure of cellulose I (Tufan et al. 2025). In the XRD pattern of the CMC/GEL, this peak was shifted to 2θ = 22.3°, and it was broader and less intense compared with that of pure CMC. This indicated the reduced crystallinity of the CMC/GEL film. This was mainly due to intermolecular interactions between CMC and GEL disrupting the original crystalline structure and the ordered arrangement of polymer chains (Bahmani et al. 2025). However, the XRD patterns of the CFS/CMC/GEL films showed increasingly narrower diffraction peaks with increasing CFS content, indicating an enhancement in crystallinity. This was mainly attributed to the formation of highly crystalline calcium lactate—generated by the reaction between organic acids and metal ions such as calcium (Kiran‐Yildirim et al. 2018)—as well as the residual concentration of inorganic salts (such as phosphates, calcium carbonate, and magnesium sulfate) from the culture medium (Hayek et al. 2019).

### 4.3 Color, Optical Properties, and Water Vapor Transmission

**FIGURE 4:**
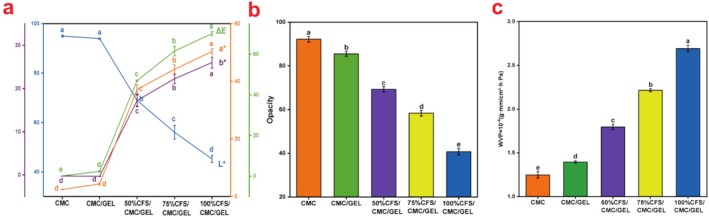
Color changes (a), light transmittance (b), and WVP (c) of the CMC, CMC/GEL, 50% CFS/CMC/GEL, 75% CFS/CMC/GEL, and 100% CFS/CMC/GEL films.

The color changes in the films were evaluated using L*, a*, b*, and Δ *E*. As shown in Figure 4a, the addition of CFS caused significant (*p* < 0.05) changes in the optical and color characteristics of the films. The L* and a* values of the CMC film and the CMC/GEL film did not show any significant differences, and the increase in the b* value was primarily attributed to the initial color of the gelatin (Asiamah et al. 2024). With increasing CFS concentration, the color of the CFS/CMC/GEL films gradually changed, as indicated by the continuous decline in the L* value along with stepwise increases in the a* and b* values. Visually, the films appeared darker, redder, and yellower as the CFS concentration was raised, resulting in an increasing total color difference (Δ *E*). Furthermore, as displayed in Figure 4b, the addition of CFS reduced the light transmittance of the films at 600 nm, indicating enhanced light barrier properties. This was due to the yellow‐brown color of CFS improving the light‐shielding capacity and color performance of the CFS/CMC/GEL films (Gözde 2024).

As shown in Figure 4c, the WVP of the pure CMC film was 1.248 ± 0.038 g/m·s·Pa × 10^−4^. The CMC/GEL film showed an improved WVP of 1.395 ± 0.013 g/m·s·Pa × 10^−4^, which was mainly due to the high water absorption capacity of gelatin (Akhtar et al. 2018). Moreover, the 100% CFS/CMC/GEL composite film achieved a further increase to 2.692 ± 0.018 g/m·s·Pa × 10^−4^. This was because the hydrophilicity of the residual sugars in CFS further enhanced the hygroscopicity of the CFS/CMC/GEL films (Shafipour‐Yordshahi et al. 2020).

### 4.4 Bacteriostasis

**FIGURE 5:**
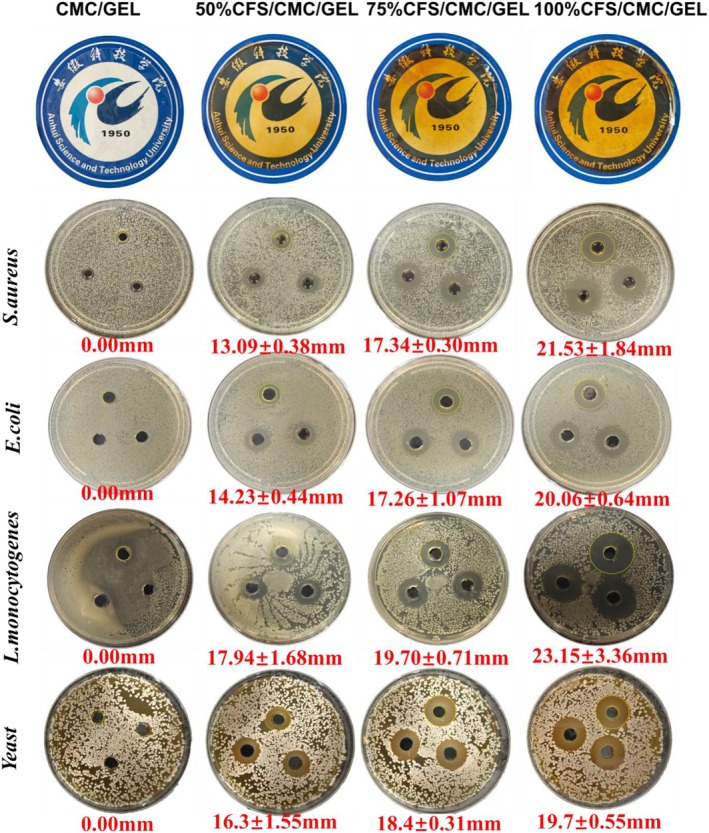
Photographs of each spraying solution (a) and antibacterial activity against *Staphylococcus aureus* , *Escherichia coli* , *Listeria monocytogenes* , and *yeast* (b).

The 50%, 75%, and 100% CFS/CMC/GEL spray solutions exhibited clear antimicrobial activity against *Escherichia coli* , *Staphylococcus aureus* , *Listeria monocytogenes* , and *yeast*, whereas the CMC/GEL spray did not show any antimicrobial activity. As shown in Figure 5, the 50%, 75%, and 100% CFS/CMC/GEL spray solutions generated 14.23 ± 0.44, 17.26 ± 1.07, and 20.06 ± 0.64 mm inhibition zones for *Escherichia coli* , respectively. For *Staphylococcus aureus* , the corresponding inhibition zones were 13.09 ± 0.38, 17.34 ± 0.30, and 21.53 ± 1.84 mm; for *Listeria monocytogenes* , inhibition zones of 17.94 ± 1.68, 19.70 ± 0.71, and 23.15 ± 3.36 mm were achieved; and for *yeast*, the inhibition zones were 16.3 ± 1.55, 18.4 ± 0.3, and 19.7 ± 0.56 mm, respectively. Raising the CFS concentration significantly enlarged the inhibition zones, demonstrating the clear concentration dependence of the antimicrobial effect (Xi et al. 2025). This trend likely reflects the greater release of bioactive constituents, including organic acids, fatty acids, hydrogen peroxide, and phenolics, with increasing CFS concentration. These bioactive substances can disrupt bacterial membranes and induce the leakage of cellular contents (Coelho et al. 2022).

## 5 Preservation of Blueberries

### 5.1 Sensory Evaluation, Decay Rate, Weight Loss, and Firmness Changes of Blueberries During Storage

**FIGURE 6:**
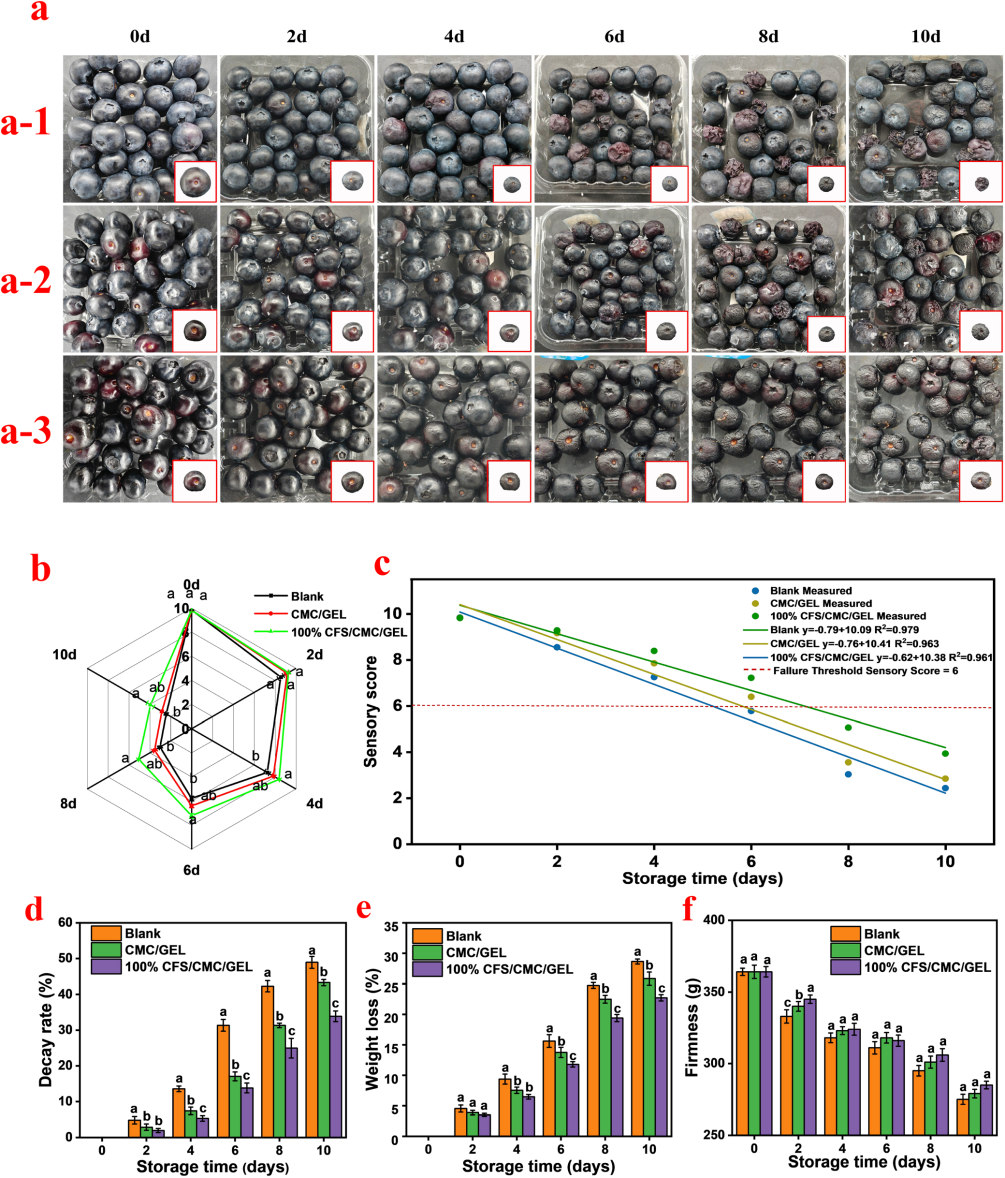
Changes in the appearance of blueberries treated with distilled water (a‐1), CMC/GEL (a‐2), and 100% CFS/CMC/GEL (a‐3). Sensory evaluation (b), zero‐order kinetic fitting (c), decay rate (d), weight loss (e), and firmness (f) during blueberry storage. Values labeled with different lowercase letters (a–c) significantly differed within the same day (*p* < 0.05).

Sensory evaluation is an intuitive indicator for evaluating the quality of fruits during storage. As shown in Figure 6a,b, the sensory quality of the blueberries gradually declined with prolonged storage time. However, the group treated with 100% CFS/CMC/GEL spray consistently maintained a relatively high sensory score, significantly outperforming the blank group (treated with distilled water). Zero‐order kinetic fitting was applied to establish a kinetic model of sensory degradation. As shown in Figure 6c, the scatter points represent measured values, the straight lines represent model fitting curves, and the red dash‐dot line represents a sensory score of 6 (the acceptability limit for shelf life). *R* ^2^ values ranging from 0.961 to 0.979 were obtained, indicating the suitability of this model for describing the sensory quality changes of the blueberries during storage. Compared with the control group, both the CMC/GEL and 100% CFS/CMC/GEL groups showed improved sensory qualities. However, the 100% CFS/CMC/GEL treatment group exhibited the best preservation effect and extended the shelf life by about 2 days compared to the CMC/GEL group. As shown in Figure 6d, the decay rate increased with increasing storage time. The 100% CFS/CMC/GEL group had the lowest decay rate (34.90%) compared with the blank (48.94%) and CMC/GEL (43.31%) groups. In the CMC/GEL group, the decay rate slowly increased before the first day but sharply rose after the sixth day, which was potentially due to the absence of antimicrobial activity in this coating as well as the retention of moisture between berries. By contrast, the phenolic compounds and organic acids (lactic, acetic, and citric acid) in CFS inhibited bacterial and *yeast* growth. It should also be noted that spraying avoids the mechanical damage caused by squeezing and friction during immersion, enabling the structure of the fruit to be preserved. Moreover, the coating acted as a physical barrier to reduce fungal invasion (Huang et al. 2023).

The overall quality of the blueberries decreased during storage, as shown in Figure 6e. However, compared with the blank group and CMC/GEL group, the 100% CFS/CMC/GEL group had significantly lower weight loss. After storage for 10 days, the 100% CFS/CMC/GEL, CMC/GEL, and blank groups had weight loss rates of 22.71% ± 0.41%, 25.85% ± 1.07%, and 28.65% ± 0.51%, respectively. The reduced weight loss of the 100% CFS/CMC/GEL group was due to the organic acids in the CFS inhibiting respiratory enzyme activity to reduce nutrient loss, while the rich antioxidants of CFS scavenged ROS to protect cell membrane integrity (Qi et al. 2020; Sharafi et al. 2024). The firmness of fresh fruit is a key factor influencing consumer preference. As shown in Figure 6f, at the end of storage, the blank, CMC/GEL, and 100% CFS/CMC/GEL groups had firmness values of 275 ± 3.48, 279 ± 2.97, and 285 ± 2.66 g. No significant difference in firmness was observed between the 100% CFS/CMC/GEL and control groups (*p* < 0.05), which is consistent with previous studies (Lee et al. 2024). In summary, the 100% CFS/CMC/GEL group outperformed the blank and CMC/GEL groups in reducing the decay rate and weight loss of blueberries during storage, offering an effective method for blueberry preservation.

### 5.2 Changes in Titratable Acidity, Malondialdehyde, and Soluble Solids of Blueberries During Storage

**FIGURE 7:**
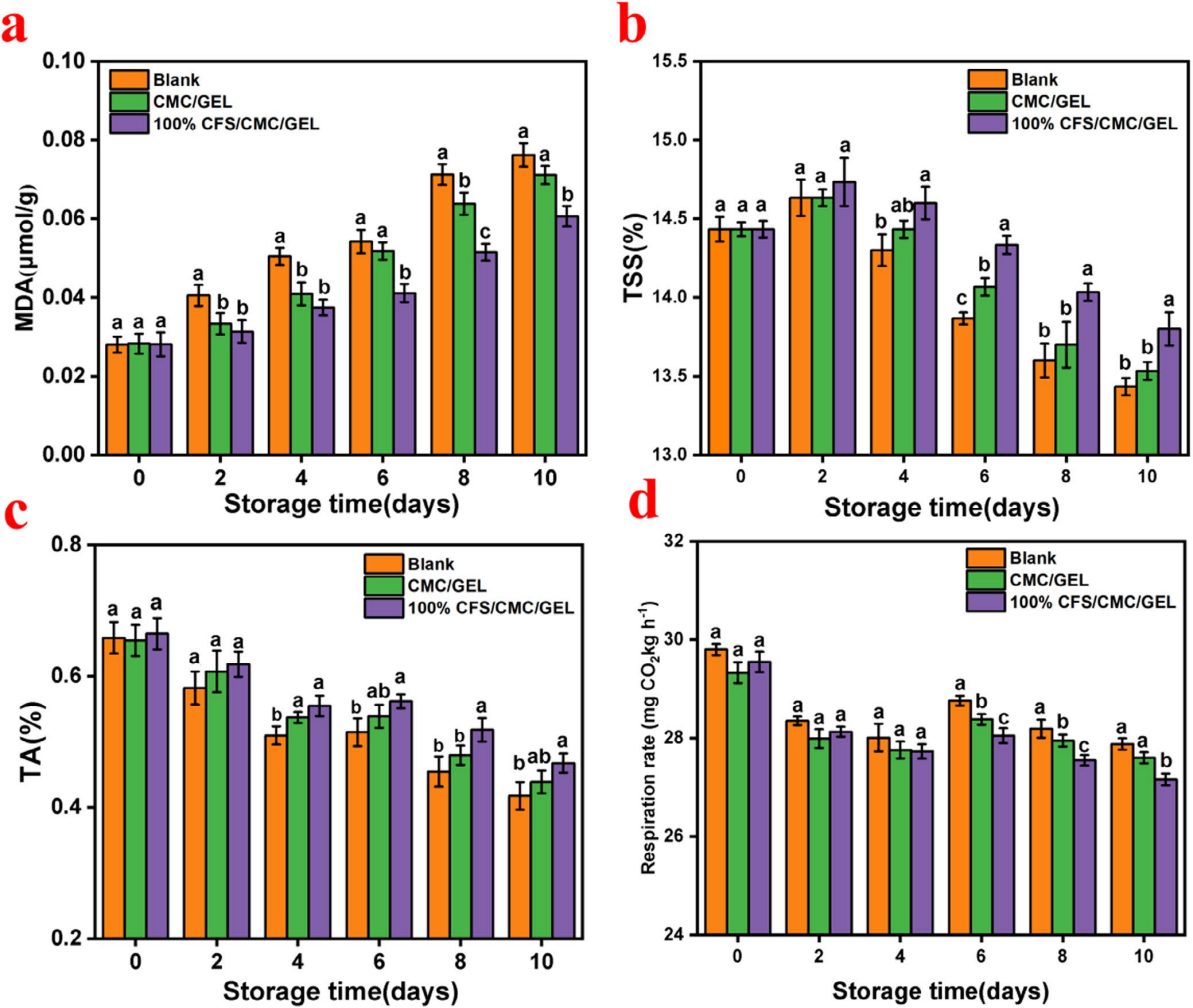
Changes in titratable acidity (a), total soluble solids content (b), and malondialdehyde (c) levels and the respiration rate (d) of blueberries during storage. Values labeled with different lowercase letters (a–c) significantly differed within the same day (*p* < 0.05).

The TA, TSS, and MDA levels of blueberries are widely recognized as indicators of fruit quality, ripeness, and membrane lipid peroxidation. As shown in Figure 7a, on day 10, the MDA levels of the blank control, CMC/GEL, and 100% CFS/CMC/GEL groups were 0.076, 0.070, and 0.061 μmol g^−1^. Therefore, treatment with 100% CFS/CMC/GEL resulted in reductions of 19.7% and 12.9% compared with the blank and CMC/GEL groups, respectively, indicating that CFS mitigated MDA accumulation. During storage, the generation of ROS leads to lipid peroxidation and the formation of MDA, which attacks the polyunsaturated fatty acids of cell membranes, exacerbating cellular damage (Liu et al. 2021).

As shown in Figure 7b, the TSS content of the blank, CMC/GEL, and 100% CFS/CMC/GEL groups increased by 0.2%, 0.2%, and 0.3%, respectively. This early rise likely reflected postharvest ripening, with polysaccharides enzymatically hydrolyzed to soluble sugars while respiratory consumption lagged behind sugar production (Abhirami et al. 2020). On Day 10, the TSS of the 100% CFS/CMC/GEL group decreased from 14.73% ± 0.10% to 13.80% ± 0.04% (the smallest decline), whereas the blank dropped from 14.63% to 13.40% (the largest decline). This was ascribed to the polyphenols in CFS scavenging free radicals and inhibiting senescence‐related processes such as lipid peroxidation and protein oxidation. Additionally, the organic acids in CFS could potentially inhibit the activity of respiratory enzymes, reducing the respiration rate to maintain a higher TSS (Sharafi et al. 2024).

As shown in Figure 7c, TA followed a “decline–slight rebound–decline” across the evaluated groups. From Day 0 to Day 4, the decline in TA was attributed to the consumption of some organic acids via respiration and the conversion of others to sugars. During this period, the TA of the 100% CFS/CMC/GEL group decreased from 0.66% ± 0.02% to 0.56% ± 0.01% (the smallest decline), while that of the blank dropped to 0.51% ± 0.01% (significantly different from 100% CFS/CMC/GEL, *p* < 0.05), with CMC/GEL showing an intermediate value. The transient increase on Day 6 potentially reflected continued postharvest metabolism and short‐term acid accumulation (Tkacz et al. 2020). On Day 8, TA declined again in all groups. Overall, TA remained higher in the 100% CFS/CMC/GEL group, which was plausibly due to the postbiotic components of this composite film delaying respiration through enzyme inhibition and slowing citrate degradation (Zhang et al. 2022), leading to an extended shelf life.

The respiration rate of postharvest fruits, which is a key factor driving their metabolism, directly determines their storage and transportation quality and shelf life (Zhang et al. 2019). As shown in Figure 7d, the respiration rates of all groups showed a downward trend during the early storage period, and a respiratory peak appeared on Day 6. The peak respiration rate of the 100% CFS/CMC/GEL group on Day 6 was 2.38% and 1.41% lower than that of the control and CMC/GEL groups, respectively. From Day 6 onward, the respiration rate of the 100% CFS/CMC/GEL group remained lower than that of the other groups. This was attributed to the low microenvironment pH provided by 100% CFS/CMC/GEL, which effectively inhibited the activity of key respiratory enzymes such as hexokinase, phosphohexose isomerase, and succinate dehydrogenase (Zhang et al. 2019). Oxidative stress is an important factor causing senescence and increasing the respiration rates in fruits (Kumar et al. 2017). However, the phenolic compounds in CFS (such as phenyl compounds like 2,4‐di‐tert‐butylphenol) can scavenge ROS, helping to reduce oxidative damage (Sharafi et al. 2024).

## 6 Conclusion

This study shows that the CFS/CMC/GEL functional spray exhibits significant antimicrobial activity, effectively extending the shelf life of small fruits such as blueberries. FTIR, XRD, and SEM analyses confirmed the good compatibility and structural integrity of CFS in the CMC/GEL matrix and showed that the inclusion of CFS enhanced the bioactive properties of the composite. Optical tests revealed that the CFS reduced light transmittance while improving the membrane barrier functions. Blueberry preservation experiments indicated that the 100% CFS/CMC/GEL group significantly reduced decay and weight loss while maintaining higher firmness. Moreover, the decline in the TSS of this group was only 4.2% over the storage period, lower than that in the blank and CMC/GEL groups. The MDA content of the 100% CFS/CMC/GEL group was 19.7% and 12.9% lower than that in the blank and CMC/GEL groups, indicating a reduction in lipid peroxidation and the better preservation of nutrients and acidity. Overall, the CFS/CMC/GEL spray developed in this work is a green, efficient, and safe preservation strategy with substantial industrial potential, and it is expected to be widely applied to the preservation of other small fruits.

## Author Contributions

**Sichen Li:** formal analysis, software. **Congsheng Yan:** writing – review and editing, funding acquisition. **Lanlan Wei:** writing – review and editing, writing – original draft. **Ziyi Qin:** writing – review and editing, formal analysis.

## Funding

This research was funded by the Excellent Research and Innovation Team of Anhui Province Universities in 2024 (2024AH010007), the Project of Science and Technology Innovation Team of Anhui Science and Technology University (2023KJCXTD003), the Key Projects of Anhui Provincial Department of Education (2024AH050316), the Key Research and Development Projects of Anhui Province (2023n06020011), and the Talent Foundation of Anhui Science and Technology University (SPYJ 202001).

## Conflicts of Interest

The authors declare no conflicts of interest.

## Data Availability

The data that support the findings of this study are available from the corresponding author upon reasonable request.
